# Accelerated Dystrophy and Decay of Oligodendrocyte Precursor Cells in the APP/PS1 Model of Alzheimer’s-Like Pathology

**DOI:** 10.3389/fncel.2020.575082

**Published:** 2020-12-03

**Authors:** Irene Chacon-De-La-Rocha, Gemma Fryatt, Andrea D. Rivera, Alexei Verkhratsky, Olivier Raineteau, Diego Gomez-Nicola, Arthur M. Butt

**Affiliations:** ^1^School of Pharmacy and Biomedical Sciences, Institute of Biomedical and Biomolecular Sciences, University of Portsmouth, Portsmouth, United Kingdom; ^2^School of Biological Sciences, Southampton General Hospital, University of Southampton, Portsmouth, United Kingdom; ^3^Faculty of Biology, Medicine, and Health, University of Manchester, Manchester, United Kingdom; ^4^University of Lyon, Université Claude Bernard Lyon 1, Inserm, Stem Cell and Brain Research Institute U1208, Bron, France

**Keywords:** hippocampus, myelin, OPC, oligodendrocyte progenitor cell, Alzheimer’s disease

## Abstract

Myelin disruption is a feature of natural aging and Alzheimer’s disease (AD). In the CNS, myelin is produced by oligodendrocytes, which are generated throughout life by oligodendrocyte progenitor cells (OPCs). Here, we examined age-related changes in OPCs in APP/PS1 mice, a model for AD-like pathology, compared with non-transgenic (Tg) age-matched controls. The analysis was performed in the CA1 area of the hippocampus following immunolabeling for NG2 with the nuclear dye Hoescht, to identify OPC and OPC sister cells, a measure of OPC replication. The results indicate a significant decrease in the number of OPCs at 9 months in APP/PS1 mice, compared to age-matched controls, without further decline at 14 months. Also, the number of OPC sister cells declined significantly at 14 months in APP/PS1 mice, which was not observed in age-matched controls. Notably, OPCs also displayed marked morphological changes at 14 months in APP/PS1 mice, characterized by an overall shrinkage of OPC process domains and increased process branching. The results indicate that OPC disruption is a pathological sign in the APP/PS1 mouse model of AD.

## Introduction

Alzheimer’s disease (AD) is the most common type of dementia and it is characterized by the formation of intracellular neurofibrillary tangles (NFTs) and extracellular amyloid-β (Aβ) plaques (Braak and Braak, 1991). White matter disruption is present at an early stage of AD pathology (Ihara et al., 2010; Bartzokis, 2011), and post-mortem analyses indicate that a loss of oligodendrocytes in AD could serve as a diagnostic tool for differentiating white matter pathologies in dementia (Sjöbeck and Englund, 2003; Brickman et al., 2015). Studies in human AD and mouse models indicate loss of oligodendrocytes and demyelination is most pronounced at the core of Aβ plaques (Mitew et al., 2010). Hence, myelin loss is a feature of human AD and mouse models (Desai et al., 2009), but the underlying causes are unresolved.

In the adult brain, oligodendrocyte progenitor cells (OPCs) are responsible for the life-long generation of oligodendrocytes, required to myelinate new connections formed in response to new life experiences, and to replace myelin lost in pathology (Young et al., 2013; McKenzie et al., 2014; Xiao et al., 2016; Hughes et al., 2018). OPCs are identified by their expression of the NG2 proteoglycan and are sometimes known as NG2-cells or NG2-glia (Butt et al., 2002). Before differentiating into mature myelinating oligodendrocytes, OPCs transition through an intermediate phase identified by expression of the G-protein coupled receptor GPR17 (Viganò et al., 2016). Notably, early changes in OPCs may be a pathological sign and underlie myelin loss in mouse models of AD-like pathology (Mitew et al., 2010; Rivera et al., 2016; Vanzulli et al., 2020). This possibility is supported by immunostaining of post-mortem AD brain showing changes in NG2 immunoreactivity in individuals with high Aβ plaque load (Nielsen et al., 2013).

The APP/PS1 transgenic mouse expresses familial AD-causing mutated forms of human APP (APPswe, Swedish familial AD-causing mutation) and presenilin1 (PS1dE9) and is used extensively as a model for AD-like pathology (Borchelt et al., 1997). The APP/PS1 mouse presents early Aβ plaque deposition in the hippocampus at 4–5 months of age and extensively throughout the forebrain by 8 months (Borchelt et al., 1997), which is linked to greatly impaired synaptic long-term potentiation (LTP) after 8 months of age in the CA1 area of the hippocampus in APP/PS1 (Gengler et al., 2010). Furthermore, several studies provide evidence that white matter and myelin disruption are early clinical signs of APP/PS1 mice (Shu et al., 2013; Wu et al., 2017; Chao et al., 2018; Dong et al., 2018), with evidence that myelin disruption in APP/PS1 mice aged 6 months is accompanied by decreased learning and spatial behavior performance (Chao et al., 2018; Dong et al., 2018). Also, there is evidence of increased NG2 cell numbers in the temporal lobe of 6 months old APP/PS1 mice (Dong et al., 2018), and clustering of hypertrophic NG2 cells around Aβ plaques in the cortex of 14-month-old APP/PS1 (Li et al., 2013). Here, we examined changes in OPCs in 9 and 14 months old APP/PS1 mice, compared to age-matched non-transgenic controls, and focused on the AD-relevant CA1 area of the hippocampus. Our results indicate a premature decline in OPC numbers at 9 months in APP/PS1, whilst at 14 months OPCs displayed cellular shrinkage and increased process branching in APP/PS1, characteristic of reactive changes in response to pathology (Ong and Levine, 1999; Butt et al., 2002). This study identifies pathological changes in OPCs in the APP/PS1 mouse model of AD.

## Materials and Methods

### Ethics

The animal study was reviewed and approved by the University of Southampton Animal Welfare Ethical Review Body (AWERB). All procedures were carried out following the Animals (Scientific Procedures) Act 1986 of the UK.

### Animals and Tissue

Transgenic APP/PS1 mice were used that contain human transgenes for both APP (KM670/671NL, Swedish) and PSEN1 (L166P). APPswe/PSEN1dE9 mice (APP/PS1) on a C57BL/6 background were originally obtained from The Jackson Laboratory and heterozygous males were bred at our local facilities with wild-type female C57BL/6J (Harlan). Offspring were ear punched and genotyped using PCR with primers specific for the APP-sequence (forward: GAATTCCGACATGA CTCAGG, reverse: GTTCTGCTGCATCTTGGACA). Mice not expressing the transgene were used as non-transgenic wild-type littermate controls. Mice were housed in groups of 4–10, under a 12-h light/12 h dark cycle at 21°C, with food and water *ad libitum*. No mice were excluded and experimental groups contained a spread of sexes. Mice weight was monitored throughout the experiment. APP/PS1 mice and age-matched non-transgenic controls aged 9 and 14 months old were perfusion-fixed intracardially under terminal anesthesia with 4% paraformaldehyde (PFA), then post-fixed for 2 h with 4% PFA. Sections were cut on a vibratome (Leica) at a thickness of 35 μm then stored in cryoprotectant at −70°C until use.

### Immunohistochemistry

Sections were treated for a blocking stage of either 10–20% normal goat serum (NGS) or normal donkey serum (NDS) or 0.5% bovine serum albumin (BSA) for 1–2 h, depending on the primary antibodies to be used. Sections were washed three times in PBS and incubated overnight in primary antibody diluted in blocking solution containing 0.25% Triton-X: rabbit anti-NG2, 1:500 (Millipore); rabbit anti-Olig2, 1:500 (Millipore); rabbit anti-GPR17, 1:100 (Cayman Labs); rat anti-MBP, 1:300 (Millipore). Sections were washed three times in PBS and incubated overnight in primary antibody diluted in blocking solution containing 0.25% Triton-X: rabbit anti-NG2, 1:500 (Millipore); rabbit anti-Olig2, 1:500 (Millipore); rabbit anti-GPR17, 1:100 (Cayman Labs); rat anti-MBP, 1:300 (Millipore). Tissues were then washed three times in PBS and incubated with an appropriate fluorochrome secondary antibody (AlexaFluor^®^ 488, AlexaFluor^®^ 568, 1:400, Life Technologies), or biotinylated secondary antibody (Vector Labs) diluted in blocking solution for 1–2 h. Finally, sections were washed three times with PBS before being mounted on glass slides and covered with mounting medium and glass coverslips ready for imaging.

### Imaging and Analysis

Immunofluorescence images were captured using a Zeiss Axiovert LSM 710 VIS40S confocal microscope and maintaining the acquisition parameters constant to allow comparison between samples within the same experiment. The acquisition of images for cell counts was done with ×20 objective. Images for OPC reconstruction were taken using ×100 objective and capturing *z*-stacks formed by 80–100 single plains with an interval of 0.3 μm. Cell counts were performed in the CA1 area in projected flattened images from *z-stacks* formed by 10 or 15 *z*-single plain images with 1 μm interval between them, and cell density was calculated as the total number of cells per unit area expressed as cells per mm^2^. The relative density of MBP immunolabeling was measured within a constant field of view (FOV) using ImageJ. For DAB immunostaining of Olig2+ oligodendrocytes, sections were examined on an Olympus dotSlide digital slide scanning system based on a BX51 microscope stand with an integrated scanning stage and Olympus CC12 color camera. The cell coverage of OPCs was measured using ImageJ by drawing a line around the cell processes and measuring the area enclosed within the line and expressing the data relative to the area of the CA1 in each section. For morphological analysis of single OPCs, cells were drawn using Neurolucida 360, and their morphology was analyzed using Neurolucida 360 explorer for measurements of the number of processes per cell, number of process terminals (end-points), number of nodes (branch points), and cell complexity; OPC cell complexity refers to the normalization and comparison of processes derived from the dendritic complexity index (Pillai et al., 2012), whereby Neurolucida 360 Explorer calculated cell *complexity* from the sum of (*terminal orders* + the *number of terminals*) multiplied by the (*total dendritic length*/*number of primary dendrites*), where the *terminal* is defined as a process ending and *terminal order* is the number of branches along a process, between the cell body and the terminal (calculated for each terminal). For Sholl analysis, the interval between Sholl shells was 5 μm. Data were expressed as Mean ± SEM and tested for significance by ANOVA followed by Tukey’s *post hoc* test for cell numbers, myelin immunostaining, OPC cell domains, and neurolucida analyses of OPCs, and Sidak’s multiple comparisons test for Sholl analysis, using GraphPad Prism 6.0.

## Results

### Premature Decline of OPCs in the Hippocampus of APP/PS1 Mice

**Figure 1:**
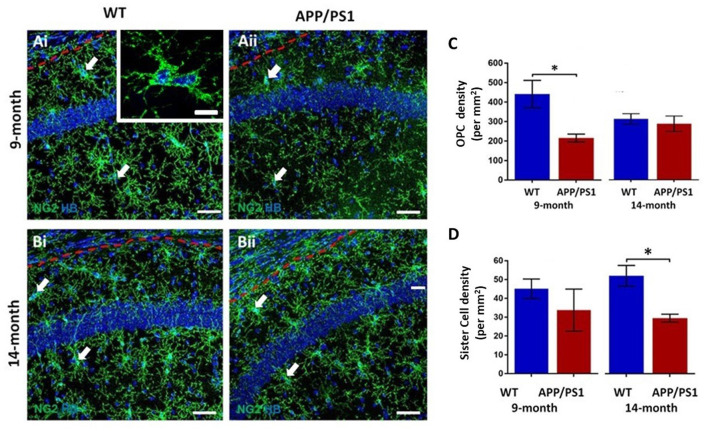
Changes in oligodendrocyte progenitor cells (OPCs) in the CA1 area of the hippocampus of APP/PS1 mice. Hippocampi of 9 months old and 14 months old APP/PS1 mice were compared to age-matched controls. **(Ai,Aii,Bi,Bii)** Representative confocal images of immunofluorescence labeling for NG2 (green) to identify OPCs and counterstaining with Hoechst (blue) for nuclei, to identify OPC sister cells (some indicated by arrows), as illustrated at higher magnification (inset, **Ai**), from non-transgenic controls **(Ai,Bi)** and APP/PS1 mice **(Aii,Bii)**, aged 9 months **(Ai,Aii)** and 14 months **(Bi,Bii)**; scale bars = 50 μm in main panels and 10 μm in the inset. **(C,D)** Bar graphs of the numerical density of NG2+ OPCs **(C)** and OPC sister cells **(D)**. Data are expressed as Mean ± SEM; *p ≤ 0.05, ANOVA followed by Tukey’s *post hoc* test, *n* = 3 animals for each group.

The hippocampus displays a high degree of adult oligodendrogenesis, which is important for learning and plasticity (Steadman et al., 2020). Here, we used NG2 immunolabeling to identify adult OPCs (Nishiyama et al., 2016) in the CA1 area of the hippocampus (Figure 1); NG2 is also expressed by pericytes, which are directly applied to blood vessels and readily distinguished from OPCs, which are distinguished by their complex process bearing morphology (Hamilton et al., 2010). OPCs are uniformly distributed throughout the hippocampus at both 9 and 14 months, in APP/PS1 mice and age-matched controls (Figures 1Ai,Aii,Bi,Bii). NG2+ OPCs are often observed as duplets or triplets (some indicated by arrows in Figures 1Ai,Aii,Bi,Bii, and at higher magnification in the inset in Figure 1Ai). OPC duplets are recently divided sister-cells and their frequency is a measure of OPC cell division (Boda et al., 2015), confirming previous studies that adult OPCs continue to divide slowly in old age (Psachoulia et al., 2009; Young et al., 2013). Quantification confirmed a significant difference in the numerical density of NG2+ OPCs in APP/PS1 at 9 months compared to age-matched controls (Figure 1C; two-way ANOVA *p* ≤ 0.05, followed by Tukey’s *post hoc* test). The data indicated a 50% decrease in NG2+ OPCs at 9 months in APP/PS1 to a level observed at 14 months in natural aging (Figure 1C); there was no further decline in OPC numbers between 9 and 14 months APP/PS1 mice, which were the same as age-matched controls (Figure 1C). Also, there was a significant decrease in the numerical density of OPC sister cells at 14 months in APP/PS1 mice (Figure 1D; two-way ANOVA *p* ≤ 0.05, followed by Tukey’s *post hoc* test, *p* ≤ 0.05). Overall, the results indicate a premature decline in OPC numbers at 9 months in APP/PS1 mice.

### Decline in Myelination in the Hippocampus of APP/PS1 Mice

**Figure 2:**
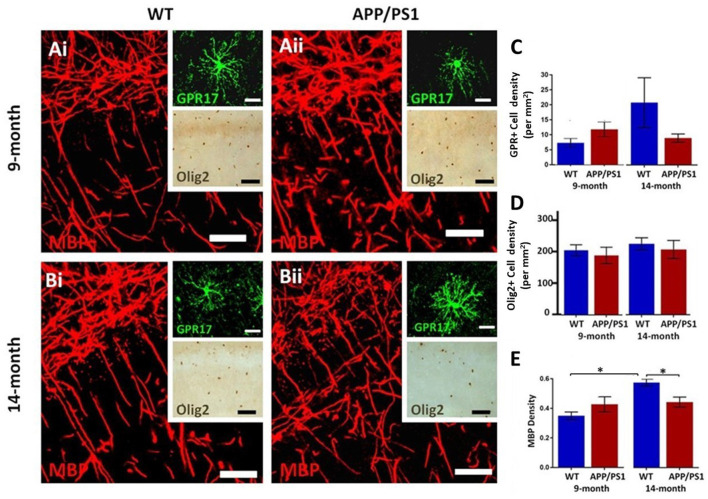
Changes in oligodendrocytes and myelin in the CA1 area of the hippocampus of APP/PS1 mice. Hippocampi of 9 months old **(Ai,Bi)** and 14 months old **(Bi,Bii)** APP/PS1 mice **(Ai,Bi)** were compared to age-matched controls **(Aii,Bii)**. **(Ai,Aii,Bi,Bii)** Representative photomicrographs of immunolabeling for MBP (red in **Ai,Aii,Bi,Bii** main panels) to identify the extent of myelination, together with GPR17 for immature oligodendrocytes (green in upper insets in **Ai,Aii,Bi,Bii**) and Olig2 for the total number of oligodendrocyte lineage cells (brown in lower insets in **Ai,Aii,Bi,Bii**); scale bars = 50 μm, except upper insets = 20 μm. **(C–E)** Bar graphs of numerical density of GPR17+ cells **(C)** and Olig2+ cells **(D)**, together with MBP immunofluorescence density **(E)**; data are expressed as Mean ± SEM; *p ≤ 0.05 ANOVA followed by Tukey’s *post hoc* test, *n* = 3 animals for each group.

The hippocampus displays a high degree of myelination, which is essential for cognitive function (Abrahám et al., 2010), and myelination has been shown to be disrupted in APP/PS1 mice, which is relevant to AD pathology (Ota et al., 2019; Chao et al., 2018; Dong et al., 2018). Immunolabeling for MBP is prominent in the CA1 area at both 9 and 14 months in controls and in APP/PS1 (Figures 2Ai,Aii,Bi,Bii), as are GPR17+ cells, which are an intermediate stage between OPCs and myelinating oligodendrocytes (upper insets, Figures 2Ai,Aii,Bi,Bii), and Olig2+ cells, which is expressed by all oligodendroglial cells (lower insets, Figures 2Ai,Aii,Bi,Bii). Between 9 and 14 months of age, we observed no significant changes in the numerical density of GPR17+ and Olig2+ oligodendrocytes, in controls or APP/PS1 (Figures 2C,D), and so we did not analyze oligodendrocyte cell numbers further; it should be noted there was wide variability in GPR17+ cells at 14 months in controls, but overall there was no apparent difference in the number of GPR17+ cells in APP/PS1 between 9 and 14 months in the CA1 region of the hippocampus. Significant age-related changes in MBP immunostaining were detected in the CA1 region and this was not observed in APP/PS1 mice (Figure 2E; ANOVA, *p* ≤ 0.01, followed by Tukey’s post hoc tests). Overall, the results indicate MBP immunostaining is retarded at later stages of pathology in APP/PS1 mice.

### OPC Exhibit Cellular Shrinkage at 14 Months in APP/PS1 Mice

**Figure 3:**
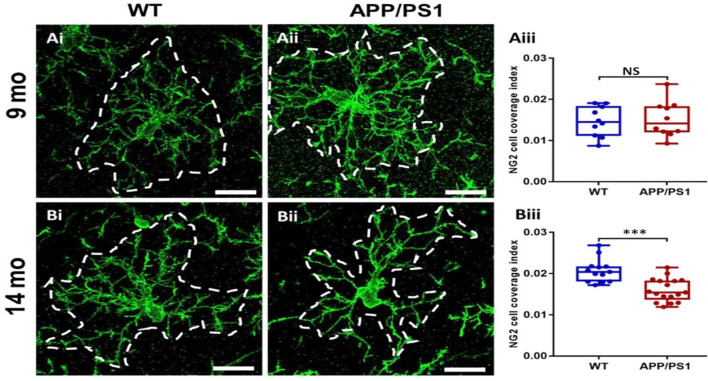
OPC process domains in the CA1 area of the hippocampus of APP/PS1 mice. Hippocampi of 9 months old and 14 months old APP/PS1 mice were examined, compared to age-matched controls, using immunofluorescence labeling for NG2 (green) to identify OPCs. High magnification confocal projections of OPCs and their process domains (indicated by broken white lines) in the 9 months old hippocampus **(Ai,Aii)**, and the 14 months old hippocampus **(Bi,Bii)**, in controls **(Ai,Bi)** and APP/PS1 **(Aii,Bii)**. Scale bars = 20 μm. **(Aiii,Biii)** Box-Whisker plots of the total area of OPC process domains. Data are Mean ± SEM, ***p ≤ 0.001, ANOVA, followed by Tukey’s *post hoc* test; NS, not significant; *n* = 10 cells for WT-9 months and APP-9 months, *n* = 13 cells for WT-14 months and *n* = 17 cells for APP-14 months, from three animals in each group.

The results above indicate OPC are disrupted in APP/PS1 mice, which is often associated with changes in OPC morphology in AD and other pathologies (Butt et al., 2019a,b; Vanzulli et al., 2020). Therefore, we examined OPC morphology in-depth, using high magnification confocal images and measuring the process domains of individual cells and the total coverage of NG2 cells within the CA1 (Figure 3). Significant differences were detected in the size of OPC process domains in 14 months APP/PS1 (Figure 3Biii; ANOVA, *p* ≤ 0.001, followed by Tukey’s *post hoc* test, *p* ≤ 0.001); no differences were observed in OPCs at 9 months in APP/PS1 compared to controls. The results indicate that at 14 months OPCs display a significant shrinkage in APP/PS1.

### OPC Exhibit Increased Process Branching and Cellular Complexity at 14 Months in APP/PS1 Mice

**Figure 4:**
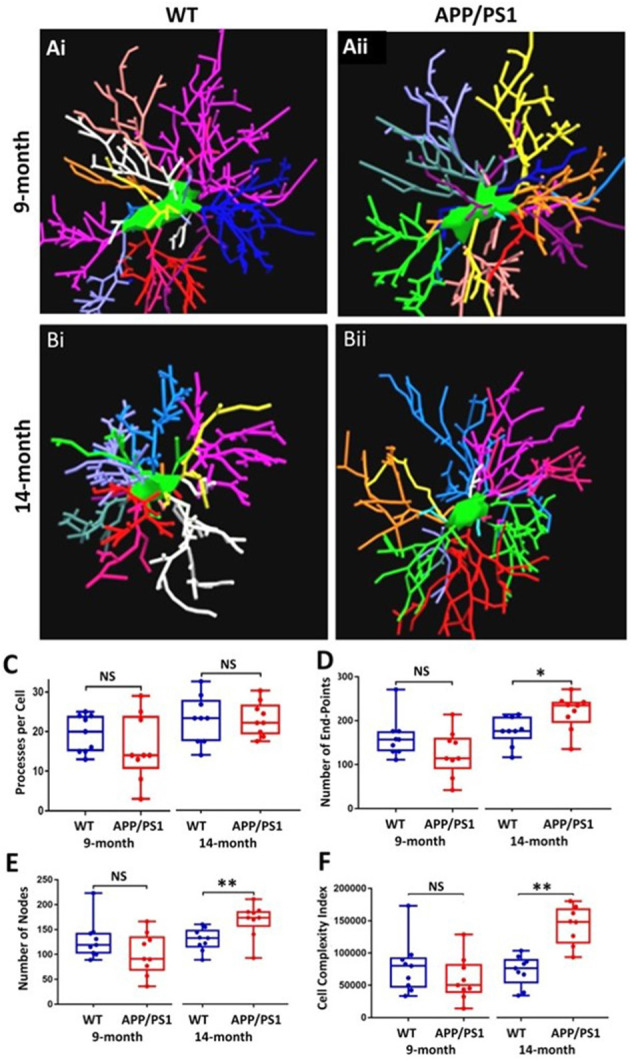
OPC morphological changes in the CA1 of the APP/PS1 mouse model compared to an age-matched controls. Data were generated by Neurolucida 360 analysis of NG2 immunostained cells **(Ai,Aii,Bi,Bii)**. Box-whisker plots of **(C)** processes per cell, **(D)** number of end-points, **(E)** number of nodes, **(F)** cell complexity index. Data expressed as Mean ± SEM. ANOVA, followed by Tukey’s *post hoc* test, *p ≤ 0.05, **p ≤ 0.01; NS, not significant; *n* = 9 cells from three animals in each group.

**Figure 5:**
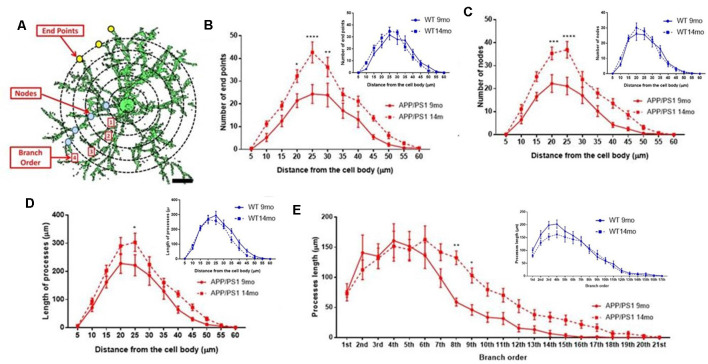
Sholl analysis of age-related changes in OPC morphology in APP/PS1 and age-matched controls. **(A)** 3D morphology of NG2 immunolabeled OPC in the CA1 area of the hippocampus (generated using isosurface rendering with Volocity software, PerkinElmer), illustrating Sholl shells (concentric circles, 5 μm apart, with the cell body in the middle), and the morphological parameters measured; the points of process branching are termed nodes (blue dots), the points where the processes intersect the Sholl shells are termed intersections (yellow dots), the number of process terminals or endpoints, and the process branch order, with 1st order closest to the cell body (adapted from Sholl, 1953, and Rietveld et al., 2015). **(B–E)** Graphs comparing OPC morphological parameters in AAP/PS1 mice aged 9 months (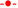) and 14 months (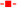), together with age-matched controls (insets); two-way ANOVA followed by Sidak’s multiple comparisons test. *p ≤ 0.05, **p ≤ 0.01, ***p ≤ 0.001, ****p ≤ 0.0001, *n* = 9 cells from three animals in each group.

The underlying morphological changes resulting in OPC shrinkage in APP/PS1 mice were examined in further detail using Neurolucida cell tracing. Confocal images of 80–100 *z*-sections, each of 0.3 μm thickness, were captured using an x100 oil objective and reconstructed and analyzed using Neurolucida 360 and Neurolucida 360 Explorer (Figures 4Ai,Aii,Bi,Bii; *n* = 9 cells from three animals in each group). Consistent with the results above, OPC morphology was significantly altered at 14 months in APP/PS1 compared to age-matched controls, with the average number of processes per cell being unaltered (Figure 4C), whereas processes displayed increased branching, with a significantly greater number of process terminals or endpoints (Figure 4D; ANOVA *p* ≤ 0.01, followed by Tukey’s *post hoc* test, *p* ≤ 0.05) and several branch points or nodes (Figure 4E; ANOVA *p* ≤ 0.01, followed by Tukey’s *post hoc* test, *p* ≤ 0.01), with a consequent 3-fold increase in the Neurolucida measurement of cell complexity in 14 month APP/PS1 compared to age-match controls (Figure 4F; ANOVA *p* ≤ 0.01, followed by Tukey’s *post hoc* test, *p* ≤ 0.01). In contrast, no changes in the morphological parameters of OPCs were detected between 9 and 14 months in wild-type mice (Figures 4C–F) or in 9 month APP/PS1 OPC compared to age-matched controls (Figures 4C–F). The age-related changes in OPC complexity in APP/PS1 mice were examined further using Sholl analysis (Figure 5A; *n* = 9 cells for each group, ANOVA followed by Sidak’s multiple comparisons test). Sholl analysis confirmed significant differences in OPC morphology in APP/PS1 mice between 9 and 14 months, with significant increases in the number of endpoints (Figure 5B), the number of nodes (Figure 5C), and inprocess lengths (Figure 5D). Also, analysis of processes length in the different branch orders identified that OPCs displayed increased process length in the distal branches (Figure 5E). In contrast to these changes in APP/PS1, no significant differences were found in OPC morphology in natural aging (Figure 5, insets); at 14 months, OPCs displayed a decrease in process lengths in the proximal branches, whereas this parameter was increased in APP/PS1 at 14 months (Figure 5E, inset). It is important to note that the small number of cells analyzed by Neurolucida and Sholl may have introduced the possibility of bias. Nonetheless, the measurements of OPC process domains, together with Neurolucida and Sholl analyses, all indicate that OPC shrinkage is a key feature in APP/PS1 at 14 months and is associated with increased process branching, giving OPCs a more fibrous appearance that is similar to “reactive” NG2 cells reported in human AD and AD models (Li et al., 2013; Nielsen et al., 2013; Vanzulli et al., 2020), as well as injury models (Ong and Levine, 1999; Butt et al., 2005; Jin et al., 2018), and this was not observed in age-matched controls.

## Discussion

Age-related loss of myelin is a pathological feature of human AD (Bartzokis, 2011; Brickman et al., 2015) and in animal models of AD (Desai et al., 2009; Mitew et al., 2010; Dong et al., 2018; Vanzulli et al., 2020). We observed a decrease in MBP immunostaining at 14 months in the hippocampus of APP/PS1 mice, consistent with evidence that myelination is disrupted in this model of AD (Shu et al., 2013; Wu et al., 2017; Chao et al., 2018; Dong et al., 2018). The key findings of the present study are that there is a premature decrease in OPC density at 9 months in APP/PS1 mice and that at 14 months OPC displayed a shrunken and fibrous morphology, indicative of morphological dystrophy. These findings indicate that changes in OPCs are potential factors in the progression of AD pathology.

Our data support previous studies that there is a decline in the number of OPCs in natural aging (Young et al., 2013). Notably, this age-related loss of OPCs occurred at 9 months of age in APP/PS1, indicating a premature loss of OPCs in this model of AD. The reduction in OPCs numbers at any point is a measure of changes in cell proliferation and/or death at earlier points, hence the reduction in OPC numbers at 9 months in APP/PS1 mice reflects an acceleration of the age-related loss of OPCs, which in natural aging occurs at later ages. The decrease in OPCs at 9 months in APP/PS1 indicates their capacity for self-renewal, defined as maintaining OPC numbers relatively constant over time, was reduced at a point before this age, which is consistent with the evidence of advanced OPC senescence in 7.5-month-old APP/PS1 mice (Zhang et al., 2019). We observed a reduction in OPC sister cells at 14 months in APP/PS1, which is a measure of recently divided OPCs (Boda et al., 2015), suggesting that OPC self-renewal may be compromised at later ages in APP/PS1, although further studies are required to confirm this, for example using multiple injections of BrdU. The changes in OPCs were associated with a reduction in MBP immunostaining at 14 months in APP/PS1 mice compared to controls. MBP immunostaining, taken as a measure of the overall extent of myelination, was increased between 9 and 14 months in wild-type controls, but not in APP/PS1 mice, consistent with multiple lines of evidence that myelination is disrupted in AD-like pathology (Desai et al., 2009; Mitew et al., 2010; Shu et al., 2013; Wu et al., 2017; Chao et al., 2018; Dong et al., 2018; Vanzulli et al., 2020). We did not detect evident changes in GPR17+ and Olig2+ oligodendrocytes, and no conclusions can be drawn on the overall numbers of oligodendrocytes at this time. The decrease in MBP immunostaining at 14 months in APP/PS1 mice may reflect changes in the number and lengths of myelin sheaths, which has been reported in aging (Hill et al., 2018; Hughes et al., 2018). Myelin remodeling is important for nervous system plasticity and repair (Chorghay et al., 2018; Williamson and Lyons, 2018; Foster et al., 2019; Ortiz et al., 2019), and the decline in myelination in APP/PS1 may be related to neuronal loss and learning dysfunction in these mice (Chao et al., 2018). The results provide evidence of OPC and myelin disruption in the hippocampus of APP/PS1 mice, suggesting key features of human AD are replicated in this mouse model.

Notably, the early loss of OPCs at 9 months in APP/PS1 hippocampus is followed at 14 months by a more fibrous appearance of NG2+ OPCs due to cell shrinkage and increased branching, similar to the fibrous morphology of “reactive” NG2-glia (Ong and Levine, 1999; Butt et al., 2002). Fibrous or reactive NG2-glia have been reported to be associated with amyloid-β plaques in human AD and mouse models (Li et al., 2013; Nielsen et al., 2013; Zhang et al., 2019; Vanzulli et al., 2020), and further studies are required to determine whether OPC morphological changes depend on their relation to amyloid-β plaques, as has been reported for astrocytes (Rodríguez et al., 2016). Since OPCs are the source of new myelinating oligodendrocytes in the adult brain (Dimou et al., 2008; Rivers et al., 2008; Zhu et al., 2008; Kang et al., 2010), it is possible their dystrophy in AD-like pathology may be a causative factor in myelin loss, but this will require comprehensive analyses to verify, using techniques such as fate-mapping and live-cell imaging. Furthermore, the underlying causes of OPC shrinkage in APP/PS1 are unresolved, but OPC are known to contact synapses in the hippocampus (Bergles et al., 2000), and reduced synaptic activity is an important feature in APP/PS1 mice (Gengler et al., 2010), which could result in retraction of OPC processes (Chacon-De-La-Rocha et al., 2020). Also, neuronal activity regulates myelination and myelin repair (Wake et al., 2011; Gibson et al., 2014; Ortiz et al., 2019), and the observed disruption of OPCs suggests this may be an important factor in myelin loss in AD-like pathology.

## Conclusion

Our findings demonstrate that OPCs undergo complex age-related changes in the hippocampus of the APP/PS1 mouse model of AD-like pathology. We conclude that OPC disruption is a pathological sign in AD and is a potential factor in accelerated myelin loss and cognitive decline.

## Data Availability Statement

The datasets presented in this article are not readily available because no datasets were generated in this study. All data generated or analyzed during this study are included in this published article. Requests to access the datasets should be directed to arthur.butt@port.ac.uk.

## Ethics Statement

The animal study was reviewed and approved by the University of Southampton Animal Welfare Ethical Review Body (AWERB). All procedures were carried out in accordance with the Animals (Scientific Procedures) Act 1986 of the UK.

## Author Contributions

IC-D-L-R: formal analysis, investigation, methodology and writing—original draft. GF: formal analysis, investigation, methodology and validation. AR: investigation. AV: conceptualization, writing—review and editing. OR: resources, writing—review and editing. DG-N: conceptualization, data curation, formal analysis, funding acquisition, project administration, resources, supervision, validation, visualization, writing—review and editing. AB: conceptualization, data curation, formal analysis, funding acquisition, project administration, resources, supervision, validation, visualization, writing—original draft, writing—review and editing. All authors contributed to the article and approved the submitted version.

## Conflict of Interest

AB and AR declare they are share-holders and co-founders of the company GliaGenesis Limited. The remaining authors declare that the research was conducted in the absence of any commercial or financial relationships that could be construed as a potential conflict of interest.
